# Kyste para testiculaire associé à un kyste rétro vésical bénin

**DOI:** 10.11604/pamj.2017.26.226.12290

**Published:** 2017-04-25

**Authors:** Adil Kallat, Ahmed Ibrahimi

**Affiliations:** 1 Service d’Urologie A, Hôpital Ibn Sina, CHU Rabat, Maroc

**Keywords:** Kyste, para testiculaire, rétro vésical, bénin, Cyst, para testicular, retro bladder benign

## Image en médecine

Il s'agit d'un patient de 34 sans antécédents particuliers et qui présente une augmentation progressive du volume scrotal avec des douleurs scrotales évoluant depuis 3 mois aggravées 5 jours avant son admission aux urgences. Par ailleurs il n'y avait pas de notion de fièvre ou de troubles du bas appareil urinaire ni de traumatisme scrotal. L'examen à l'admission retrouvait un patient en assez bon état général. L'examen des bourses trouvait un hémiscrotum droit une augmenté de volume (A) avec une douleur accentuée par la palpation et absence de signes d'inflammation locale. Par ailleurs, l'examen de l'hémiscrotum gauche était sans particularités. Le dosage des marqueurs tumoraux était normal. L'échographie du contenu scrotal associée au doppler a objectivé une masse para testiculaire droite remontant le long du cordon spermatique et se terminant en rétro vésical par une masse kystique arrondie de contours réguliers. Nous avons réalisé une orchidectomie par voie inguinale (B) avec exérèse de la masse rétro vésicale (C). L'examen anatomopathologique des deux pièces est revenu en faveur de kystes bénins.

**Figure 1:**
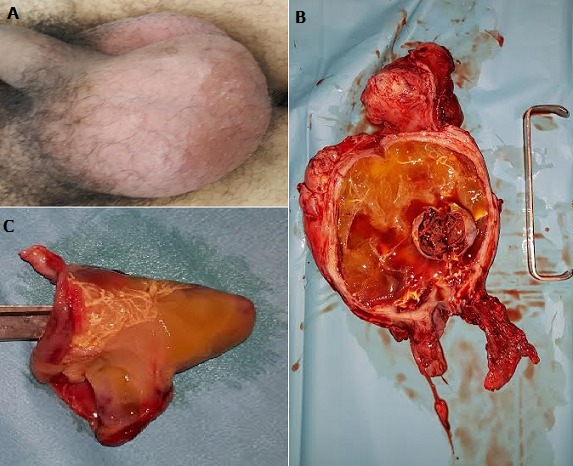
(A) hémi scrotum droit augmenté de volume; (B) pièce d’orchidectomie après son ouverture; (C) kyste rétro vésical après son ouverture et son inversement

